# Geographical Range and Local Abundance of Tree Species in China

**DOI:** 10.1371/journal.pone.0076374

**Published:** 2013-10-10

**Authors:** Haibao Ren, Richard Condit, Bin Chen, Xiangcheng Mi, Min Cao, Wanhui Ye, Zhanqing Hao, Keping Ma

**Affiliations:** 1 Key Laboratory of Vegetation and Environmental Change, Institute of Botany, Chinese Academy of Sciences, Beijing, P.R. China; 2 Smithsonian Tropical Research Institute, Panama City, Republic of Panama; 3 Xishuangbanna Tropical Botanical Garden, Chinese Academy of Sciences, Kunming, P.R. China; 4 South China Botanical Garden, Chinese Academy of Sciences, Guangzhou, P.R. China; 5 Institute of Applied Ecology, Chinese Academy of Sciences, Shenyang, P.R. China; Consiglio Nazionale delle Ricerche (CNR), Italy

## Abstract

Most studies on the geographical distribution of species have utilized a few well-known taxa in Europe and North America, with little research in China and its wide range of climate and forest types. We assembled large datasets to quantify the geographic ranges of tree species in China and to test several biogeographic hypotheses: 1) whether locally abundant species tend to be geographically widespread; 2) whether species are more abundant towards their range-centers; and 3) how abundances are correlated between sites. Local abundances of 651 species were derived from four tree plots of 20–25 ha where all individuals ≥1 cm in stem diameter were mapped and identified taxonomically. Range sizes of these species across China were then estimated from over 460,000 geo-referenced records; a Bayesian approach was used, allowing careful measures of error of each range estimate. The log-transformed range sizes had a bell-shaped distribution with a median of 703,000 km^2^, and >90% of 651 species had ranges >10^5^ km^2^. There was no relationship between local abundance and range size, and no evidence for species being more abundant towards their range-centers. Finally, species’ abundances were positively correlated between sites. The widespread nature of most tree species in China suggests few are vulnerable to global extinction, and there is no indication of the double-peril that would result if rare species also had narrow ranges.

## Introduction

Two macro-ecological patterns regarding the geographic distribution of species are widely cited. One is a positive *abundance-range* relationship, meaning a tendency for locally abundant species to be geographically widespread [1]. The second is a propensity for species to decrease in abundance from their range centers toward the range edges, known as *abundant-center* distribution [2]. These two patterns have been called ‘general rules’ in bio-geography [3], [4].

Consistent patterns or ‘rules’ in geographic ranges can contribute to an understanding of extinction risk and influence the design of conservation areas [5], [6]. For example, the positive abundance-range relationship suggests that rare species face two extinction risks at once [7], [8]: low local abundance increases the likelihood that species go extinct, due to demographic and environmental stochasticity [8], while narrow geographic range increases the chance that the whole population undergoes environmental stress simultaneously [9]. The abundant-center pattern, meantime, contributes to predictions about how the boundaries of species distributions might respond to climatic changes [3], [10].

According to reviews by Gaston and Blackburn [9] and Blackburn *et al*. [4], most of the tests of abundance-range relation showed a positive correlation, but there were exceptions, and the strength varied across regions, habitats and taxa. These studies, however, have been dominated by a few taxonomic groups such as mammals and especially birds [11]–[22], with nearly all done in North America, Europe, and Australia [3], [4], [23]. A handful of studies on trees from temperate forests have shown an inconsistent and weak abundance-range relationship [4], [23]–[26]. The abundant-center distribution has been theoretically supported by a number of mechanisms [3], [27], [28], nonetheless it has rarely been tested empirically [3], [10], [29], [30], and some recent evidence failed to detect the pattern [24], [31], [32], suggesting that the distribution of species abundance may be more complicated than was assumed previously. Our studies in China, where little prior work has been done, and on trees of subtropical forest, a very seldom-studied biome, may thus shed light on the generality of major biogeographic patterns [10]. China’s large area of subtropical, broad-leaved forest is under intense development pressure, and knowledge about abundances and geographic ranges of its native tree species should form the basis of a conservation agenda.

Recently, over 6 million Chinese herbarium records were digitized, including specimen labels, Latin names, and coordinates; and we have compiled the counties, towns or villages of species occurrences mentioned in published local flora to augment the herbarium records. At the same time, we have built a network of permanent forest dynamic plots in China, called CForBio (Chinese Forest Biodiversity Monitoring Network) [33]. Here we utilize precise estimates of local abundance for several hundred tree species, including rare ones, form four of these plots, and together with the distributional data we address a series of basic questions about abundances and geographic ranges of trees in China. 1) What are the geographic ranges, and how many species are limited to narrow areas? 2) Do species that are locally abundant tend to have wide geographic ranges? 3) Are species more abundant toward the centers of their ranges? Finally, 4) are abundances correlated site-to-site for those species which occur in more than one plot?

## Methods

### Ethics Statement

No specific permissions were required for the field studies described here at the four study locations: Changbaishan National Nature Reserve, Gutianshan National Nature Reserve, Dinghushan National Nature Reserve and Xishuangbanna National Nature Reserve. The Reserves are owned and managed by the state and its government, and the locations, including the sites for our sampling, are not privately-owned or protected in any way. The field studies did not involve endangered or protected species in these areas.

### Study Sites

**Figure 1:**
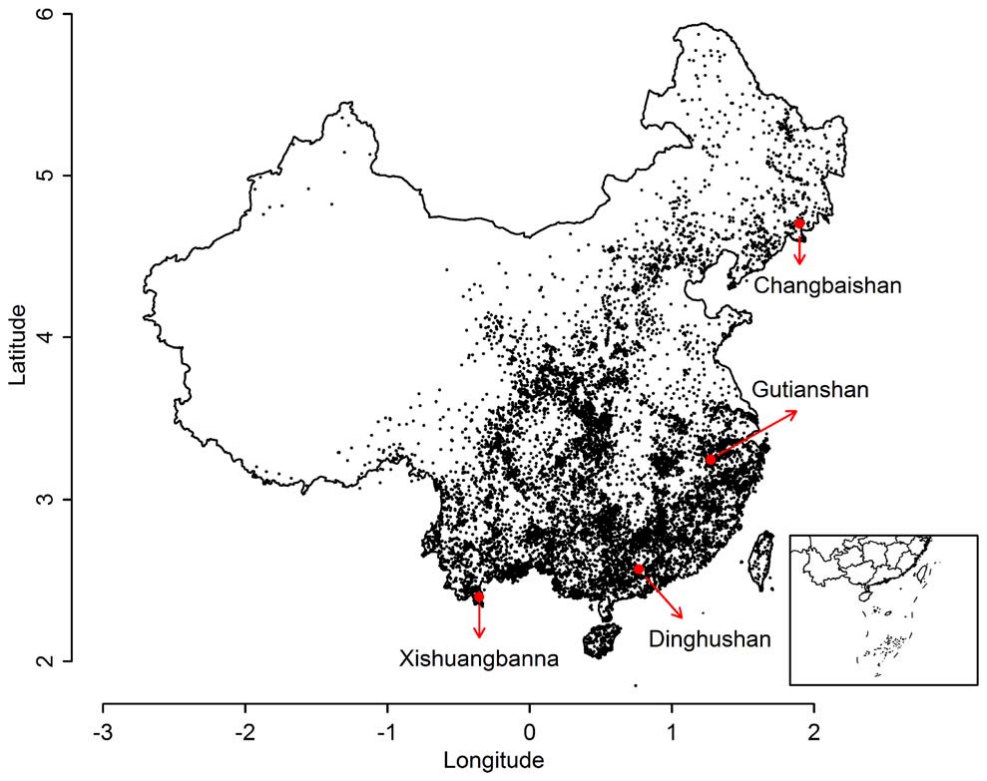
Distribution of geo-referenced records for tree species in China. Included are 461122 records of the 707 tree species occurring in the four marked plots (red dots). Units on the axes are 1000 kilometers.

The four Reserves span a latitudinal range of 21° N to 42° N (Fig. 1; Table 1). They all are in eastern China, in moist to humid forest, and span a wide temperature range [34]–[37].

**Table 1:** Size, geographic location, number of species, and endemics to China for the four census plots, plus the geo-referenced record number for those species.

Plot	Area (ha)	Longitude(E°)	Latitude (N°)	Species*	Endemic*	Geo-referenced record number#		
						Specimen	Flora	Total
Changbaishan	25	128.083	42.3833	52 (50)	1 (1)	31839 (31828)	5510 (5501)	37401 (37397)
Gutianshan	24	118.120	29.2537	159 (157)	55 (55)	210033 (210029)	14275 (14271)	224500 (224490)
Dinghushan	20	112.510	23.1558	208 (194)	35 (31)	162328 (162311)	11676 (11673)	174279 (174244)
Xishuangbanna	20	101.576	21.6138	357 (318)	58 (44)	108117 (107976)	14629 (14524)	123153 (122867)
Total	89	–	–	707 (651)	142 (124)	419829 (419658)	40510 (40389)	461122 (460773)

* The first number is the total species number, and in parenthesis the number of those species with ≥20 geo-referenced records.

# The first number is the total number of geo-referenced records over all the species, and parentheses the number of those species with ≥20 geo-referenced records; total also includes all records, specimens plus flora plus plots.

### Tree Census Plots

A large-scale forest plot (≥20-ha) was censused fully at each Reserve. All free-standing individuals ≥1 cm in stem diameter at breast height (DBH) were mapped, measured, and identified to species, following Condit [38]. In the Xishuangbanna plot, at the boundary between tropics and subtropics and near the borders of Laos, Burma and Vietnam, there were over 357 species. At the other extreme, the Changbaishan plot near the Korean border had just 52 species. The other two plots in subtropics were also quite diverse (Table 1). The pooled species list from all four plots had 707 species (excluding 106 species not identified in the Xishuangbanna plot). The latitude and longitude of each plot were collected by GPS. Assuming all species within a plot have the same geographic coordinates, the four plots provided 783 individual geo-referenced records for the 707 species.

### Specimen Records

Latin name, latitude, and longitude were available for 6 million digitized records from 43 herbaria in China [39]. We matched all records against the species names from the four plots, producing 419829 individual records of 707 species (Fig. 1).

### Local Flora

We consulted 475 published floras and noted all counties, towns, and villages in which the same 707 species occurred. The latitude and longitude of the geographic center of each location was taken from digital maps. This produced another 40510 records whose coordinates are accurate to the size of the political entities. A total of 1593 counties were involved, the median size of which was 1994.4 km^2^. This imparted an error of approximately 10^3^ km^2^ for geographic ranges, which was very small compared to estimated range sizes.

### Estimated Range Size

In all analyses, we pooled the herbarium specimens with the records from published flora and from permanent plots (Fig. 1). Latitude and longitude were then converted to kilometers, assuming 1 degree latitude is 110.95 km and 1 degree longitude at the equator is 111.32 km; at latitude *L*, a degree longitude is smaller by a factor cos(*L*). Longitude was thus converted to kilometers, using the equation.
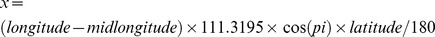
using 105° as the approximate mid-longitude of China. Latitude was converted with the equation 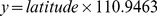. This projection resulted in a small error in estimated area at northern latitudes far from 105° longitude, but the error was <1% even in northern China. We referred to the east-west coordinates in km as *x*, and north-south as *y*.

Species are often distributed normally along an environmental gradient, especially at regional or continental scale [40], so we modeled range sizes using a bivariate Gaussian distribution [41]–[43]. We fitted the Gaussian to the distribution of each species with at least three separate records, since it is not possible with one or two records. We thus asserted that 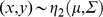, where 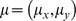, 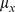 is the mean of *x*, 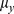 the mean of *y*; 
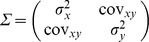
, 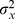 is the variance of *x*, 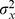 the variance of *y*, 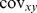 the covariance between *x* and *y*; and *η*_2_ indicates the bivariate Gaussian. We called 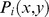 the probability that a randomly sampled individual of species *i* would be at location (*x, y*) (practically within a square kilometer around the location (*x, y*)). At the center of the estimated Gaussian distribution, the probability is maximal, 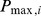. The parameters 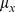, 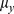, 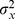, 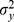 and 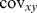 were estimated by a Bayesian approach, using a Gibbs sampler to create posterior distributions for each. Details are presented in supporting materials (Method S1), with the source code in the programming language R [44] included (Program S1).

**Figure 2:**
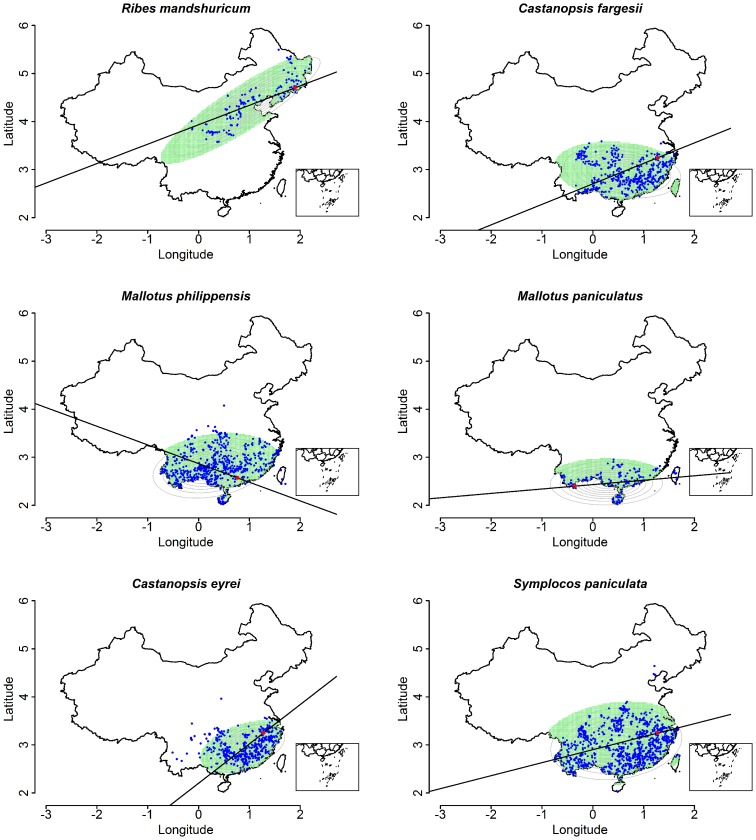
Illustrations of Gaussian distributions estimated with the Bayesian likelihood approach. Red points give locations of the plots in which each species was observed. Straight lines join each plot and the range centers. Ten probability contours evenly spaced from zero to 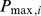 are shown in gray.

The range size of each species was then defined by dividing China into a grid of 25×25 km squares, and finding 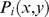 in each for every species *i*. All cells with probability 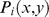 >0.05*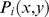 were defined as the range of species *i*, counting only cells inside China, but not including the islands in the South China Sea. The number of cells was multiplied by 625 to produce the range size in square kilometers. The fitted distributions of sample species are shown in Figure 2. Narrow endemics are defined as those with ranges <100,000 km^2^ [45].

**Figure 3:**
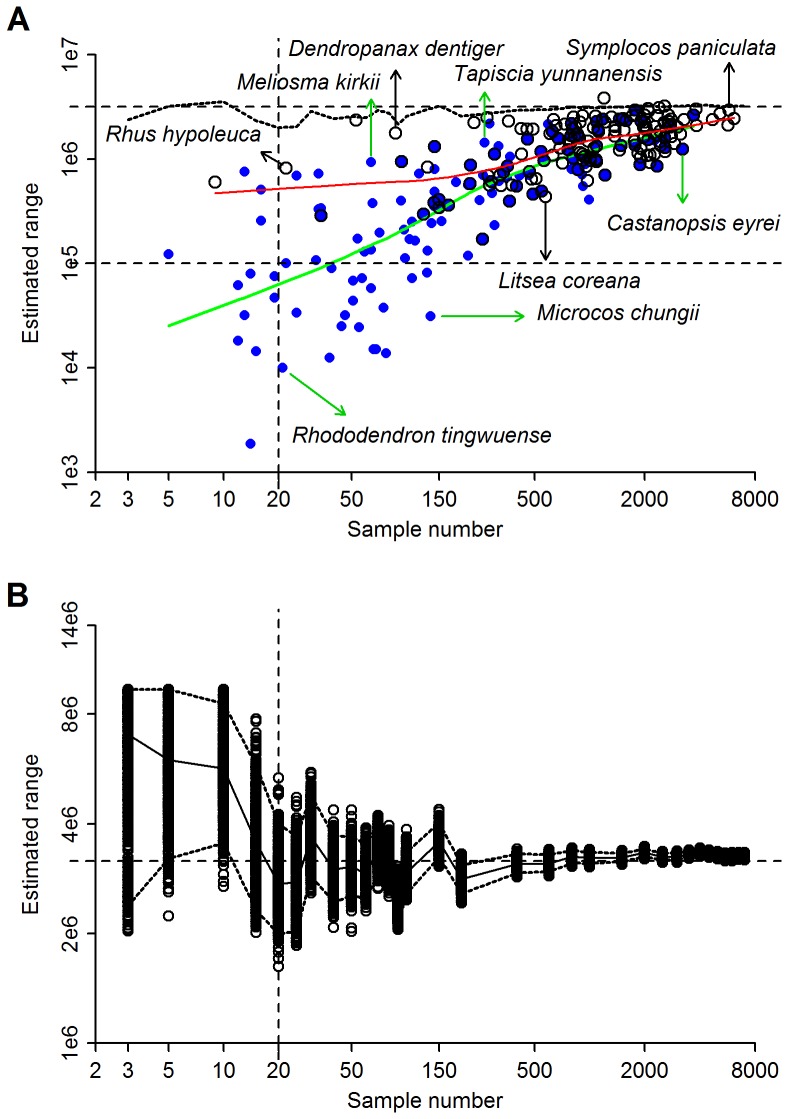
Relationship between estimated range size and sample size of geo-referenced records. Panel A is for real species, and Panel B for random draws from the entire pool of geo-referenced records. In panel A, solid points indicate species from Gutianshan; open circles are for species endemic to China (any of the plots). The green solid line (lower) is a trend curve through the Gutianshan points, and the solid red (upper) line through the endemics. The dotted curve is the lower 95% confidence limit of random ranges (from Panel B). Panel B is a test for bias in estimated range size caused by sample size. The solid, central curve is the mean range size from 1000 draws at each value of N (sample number of geo-referenced records), and two dotted curves are 95% confidence intervals (the central 95 percentiles of 1000 draws). Samples were done at *N* = 3, 5, 10, 15, 20, 25, 30, 40, 50, 60, 70, 80, 90, 100, 150, 200, 400, 600, 800, 1000, 1500, 2000, 2500, 3000, 3500, 4000, 4500, 5000, 5500, 6000, 6500, 7000.

This method was designed to describe the area over which a species was found. It does not address whether all habitats within the area are appropriate. It has the substantial advantage of discounting rare outliers in a quantitative way (there are several examples in Fig. 2 and Fig. 3), and use of the Bayesian fitting precisely described confidence in the estimated ranges. Traditional techniques to estimate range size of species, such as Polygon methods surrounding the observed points, cannot offer precise confidence limits and moreover are biased with respect to abundance. For comparison, we also estimated range sizes for each species using the Minimum Convex Polygon (MCP) estimator in R-package ‘adehabitatHR’ Version: 0.4.7 [46]. The R^2^ between range sizes estimated by the two techniques was very high up to 0.90, and the ranges of two species with few records were greatly underestimated by MCP (Figure S1).

### Estimated Range and Sample Size

To judge whether range estimates based on Gaussian fits were biased by sample size, we randomly drew *N* records from the entire pool of 461,122 coordinates (Table 1) and ran the range-fitting routine, repeating 1000 times for individual values of *N* from 3 to 7000. The random simulation indicated that the variance of estimated range decreased with increasing sample size *N*, and that the mean asymptotically reached ca. 3.3×10^6^ km^2^ with small variance for *N*≥20; below *N* = 20, estimates were unreliable (Fig. 3). We thus included in our analyses the 651 species with ≥20 occurrences.

### Plots Near China’s Border

Unfortunately, three of the four plots: Changbaishan, Dinghushan, and Xishuangbanna, are close enough to China’s borders that species there are likely to have a substantial distribution outside our records (Fig. 1). Until we gather additional data from neighboring countries to the south and north, we do not have good estimates for total range size of these species. However, subtropical humid forest in China is unique in the world, and the Gutianshan plot is in lowlands near its center (Fig. 1), so we assumed estimated ranges for species from the plot Gutianshan are near complete. In addition, we had a subset of species whose ranges were known fully: 142 species of the 707 species are endemic to China [47]. In presenting absolute measures of range size, we consider only the species from the Gutianshan plot and all endemic species.

### Abundance-range Relationship

Only full ranges were considered, so the analyses were based on all Gutianshan species, plus endemics (though with only one endemic at the Changbaishan plot, the analysis could not be done there). Local abundance of a species was measured as the number of individual trees ≥1 cm DBH in one plot. Range size and local abundance are both approximately log-normally distributed [48], [49], and were thus log_2_-transformed for plotting and a linear regression analysis.

### Abundant-center Distribution

A standard test of this hypothesis is to consider how abundance within a species varies from its range center toward the edge, but most species we examined occurred at no more than two sites. The best we can do is ask whether, in those cases, species were more abundant in the plot closer to their range center than in the other plot. We also attempt an alternative test of the abundant-center hypothesis by turning it into an inter-specific instead of intra-specific prediction: we ask whether variation in abundances among species at one site can be explained by the plot’s distance from each species’ range center. That is, we hypothesize that the most abundant species in a plot are the ones whose ranges are centered near the plot, while rare species at the same site have range centers far away. For these two tests, only species with full ranges (species from the Gutianshan plot and endemics) were considered.

The range center for species *i* was defined as the location where the probability 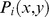 was the highest, 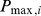, and the range edge as the location where 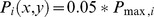. A straight line was drawn from the center of a plot to the center of the geographic range of each species *i*, and the occurrence probability 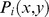 was calculated along that line every 25 km along the *x*-axis. The distances from a plot to range center (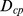) and from range edge to range center (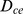) were taken along the line. We refer to the ratio 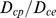 as the relative distance (*D_rcp_*) from a plot to range center; if the plot was outside the species’ range, then *D_rcp_*>1.

Of the species with full ranges, there were 32 species present in at least two plots (24 in Gutianshan and Dinghushan, five in Gutianshan and Xishuangbanna, one in Gutianshan and Dinghuashan, plus two in all three plots). For the first test, we calculated the difference in abundance between each pair of plots (species present in three plots contributed three pairs). Define the difference in abundance, relative to total abundance, as Δ*A* = (*A_2_-A_1_*)/*max*(*A_2_*-*A_1_*), and the difference in relative distance to range center, Δ*D_rcp_* = *D_rcp2_*-*D_rcp1_* (subscript 1 refers to the plot closer to the range center, 2 the one further). We used a Wilcoxon test to examine the relationship between Δ*A* and Δ*D_rcp_*. We also tried the same procedure using absolute distance from plots to species’ range-centers (*D_cp_*), instead of *D_rcp_*. For the second test, *D_rcp_* was used as the predictor in a linear regression for all 226 species with full ranges (the species from Gutianshan and all endemics), with log_2_-transformed abundance in a plot the response.

### Correlation in Abundance between Plots

The Dinghushan plot had 24 species in common with Gutianshan, and 37 with Xishuangbanna. For each set, Pearson correlation coefficients were calculated between log_2_-transformed abundances at the two sites. Other plot comparisons were not done, since the Changbaishan plot had no species in common with other sites, and there were only seven species common to the Gutianshan and Xishuangbanna plot.

## Results

### Range Sizes

**Figure 4:**
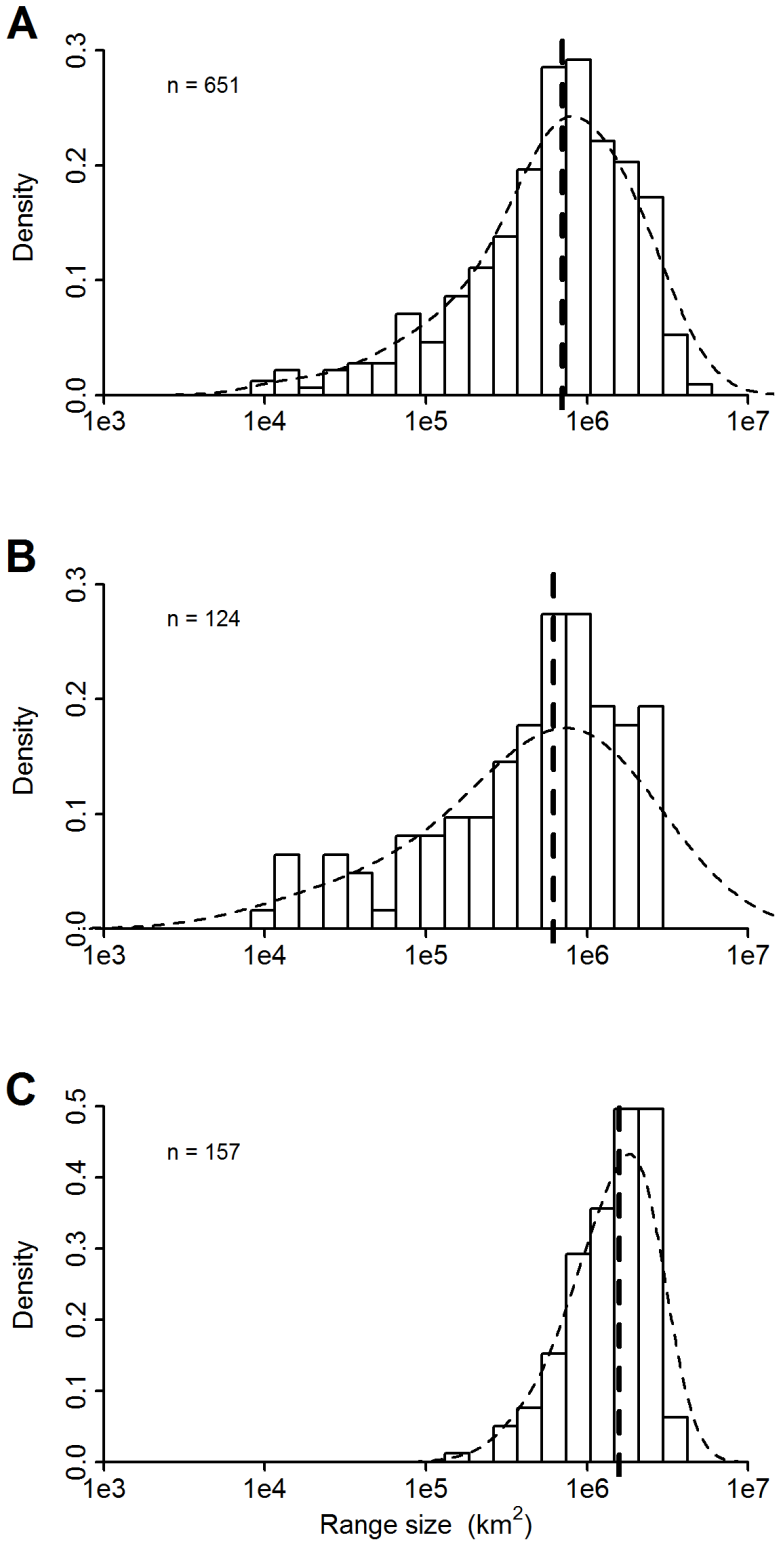
Histogram of species’ range sizes. Panel A is for all species in the study, panel B for all endemics, and Panel C for all species from the Gutianshan plot. In each case, only species with ≥20 geo-referenced records were included.

Histograms of log-range sizes were bell-shaped (Fig. 4). Median range size was 7.03×10^5^ km^2^ for all species together, 6.18×10^5^ km^2^ for all endemic species, and 1.57×10^6^ km^2^ for all species from the Gutianshan plot (Fig. 4). Of 651 tree species, 63 (9.68%) had a range <10^5^ km^2^, 18 of which were endemics. Just one species, the endemic *Aidia yunnanensis*, had a range<10^4^ km^2^. All 63 of these range-restricted species were from Xishuangbanna (61 species) and Dinghushan (the other two), close to China’s border.

**Figure 5:**
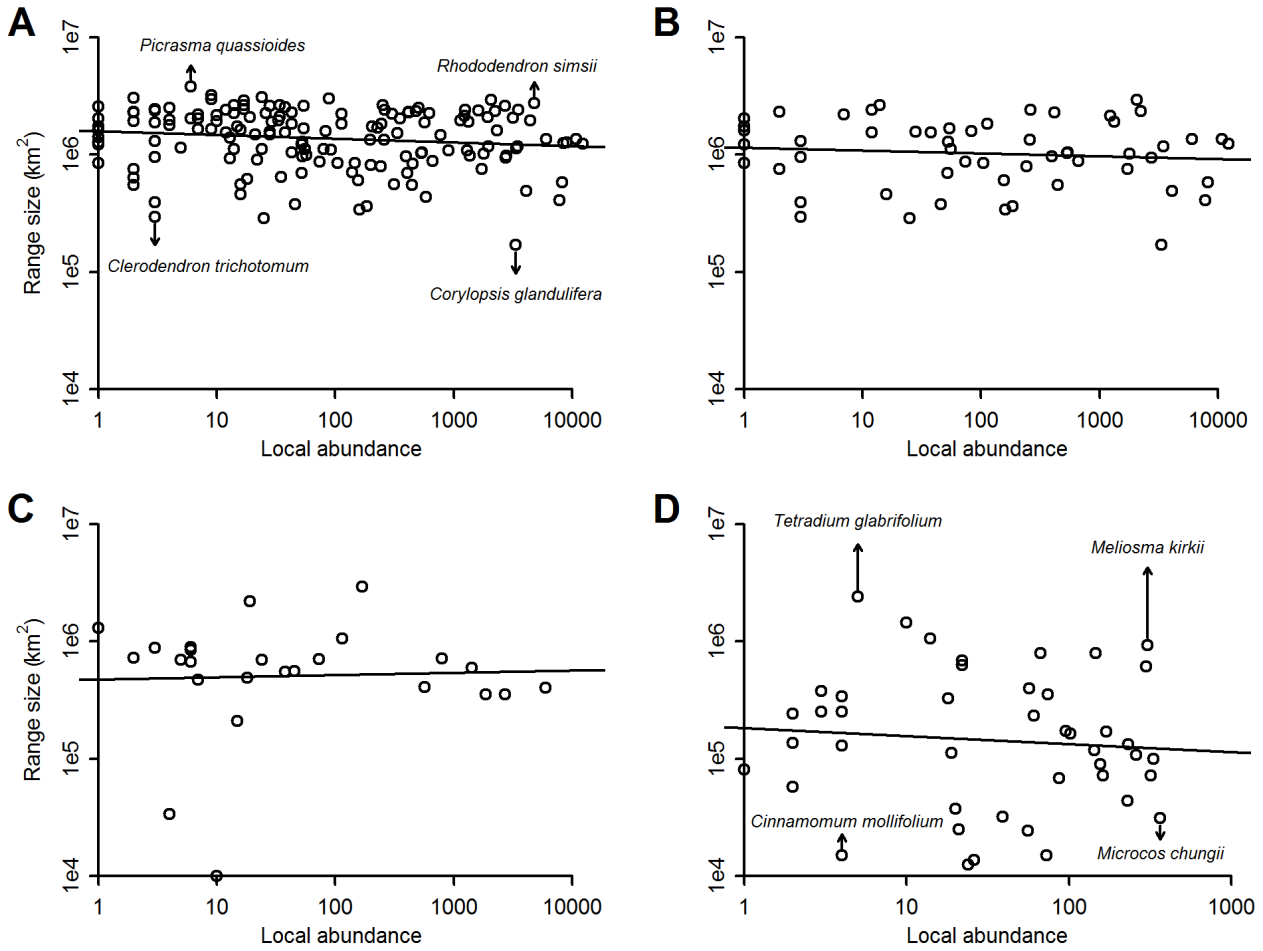
Relationship between range size and local abundance. Panel A shows all species from the Gutianshan plot, and the other panels endemics from Gutianshan (B), Dinghushan (C), and Xishuangbanna (D). The axes were both log_2_-transformed. In each case, only species with ≥20 geo-referenced records were included.

Even the Chinese endemics from Gutianshan had a median range size over one million square kilometers (1.11×10^6^), and the endemics from Dinghushan were close at a median of 6.94×10^5^ km^2^. The endemics from Xishuangbanna had smaller ranges, with median 1.34×10^5^ km^2^. Endemics from Xishuangbanna illustrated great variation in range size, for example, *Tetradium glabrifolium* had a range of 2.4×10^6^ km^2^, while *Cinnamomum mollifolium* had a range of only 1.5×10^4^ km^2^ (Fig. 5D).

### Abundance-range Relationship

Including all species at Gutianshan, the regression was just barely significant, with a negative slope, meaning that abundant species had narrower ranges (sample size = 157, intercept = 20.6, slope = −0.03, *p* = 0.078, *R^2^* = 0.02). The weakness of the relationship can be underscored by citing sample species: *Picrasma quassioides* was locally rare but geographically widespread, while *Corylopsis glandulifera* was locally abundant but geographically limited. Contrastingly, *Clerodendron trichotomum* was locally rare and geographically restricted, while *Rhododendron simsii* was locally common and broadly distributed (Fig. 5A). When only endemic species were considered, there were no significant relationships between abundance and range (Gutianshan: sample size = 55, intercept = 20.1, slope = −0.02, *p* = 0.45; Xishuangbanna: sample size = 44, intercept = 17.5, slope = −0.07, *p* = 0.58; Dinghushan: sample size = 25, intercept = 18.9, slope = 0.02, *p* = 0.85). The regression slopes were negative for the first two plots, positive for the third (Fig. 5B–D). Species with highly divergent patterns dictate the weak relationship: at Xishuangbanna, *Cinnamomum mollifolium* had a narrow distribution with low abundance, while *Meliosma kirkii* had a wide distribution with high abundance. Contrastingly, *Tetradium glabrifolium* was widely distributed with low abundance, while *Microcos chungii* was limited to a narrow area but had high local abundance.

### Abundant-center Distribution

**Figure 6:**
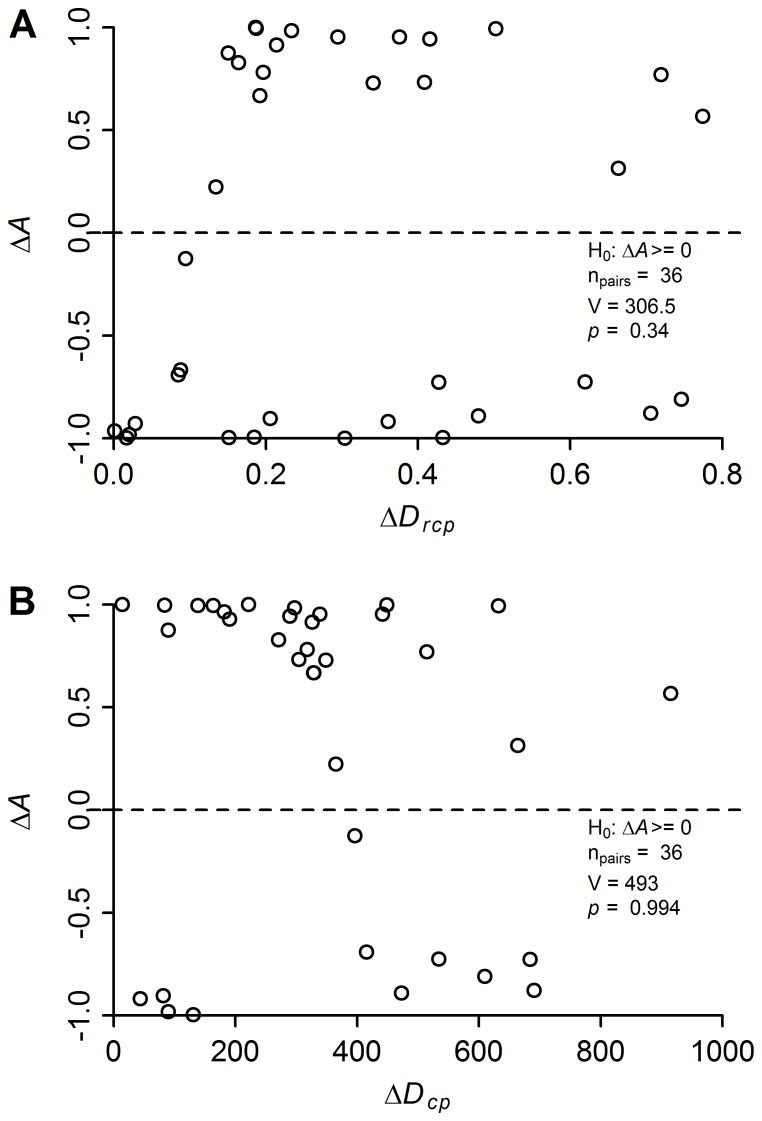
Correlation between differences in species’ abundances (Δ*A*) and differences in distances from their range centers. Panel A uses relative distances from species’ range centers (Δ*D_rcp_*), and panel B absolute distances (Δ*D_cp_*).

Abundance was higher in the plot closer to a species’ range center in exactly half of the 36 cases where a comparison between two plots was possible, and the Wilcoxon test reported no relationship, thus rejecting the hypothesis that abundance within a species decreased with increasing distances from the species’ range-center (Fig. 6A). The hypothesis was likewise rejected if we used absolute distance from range center, rather than the relative distance (Fig. 6B). In the inter-specific test of the abundant-center hypothesis, we found no relationship in abundances among species and distances from their range centers (*p* = 0.90).

### Correlations in Abundance among Plots

**Figure 7:**
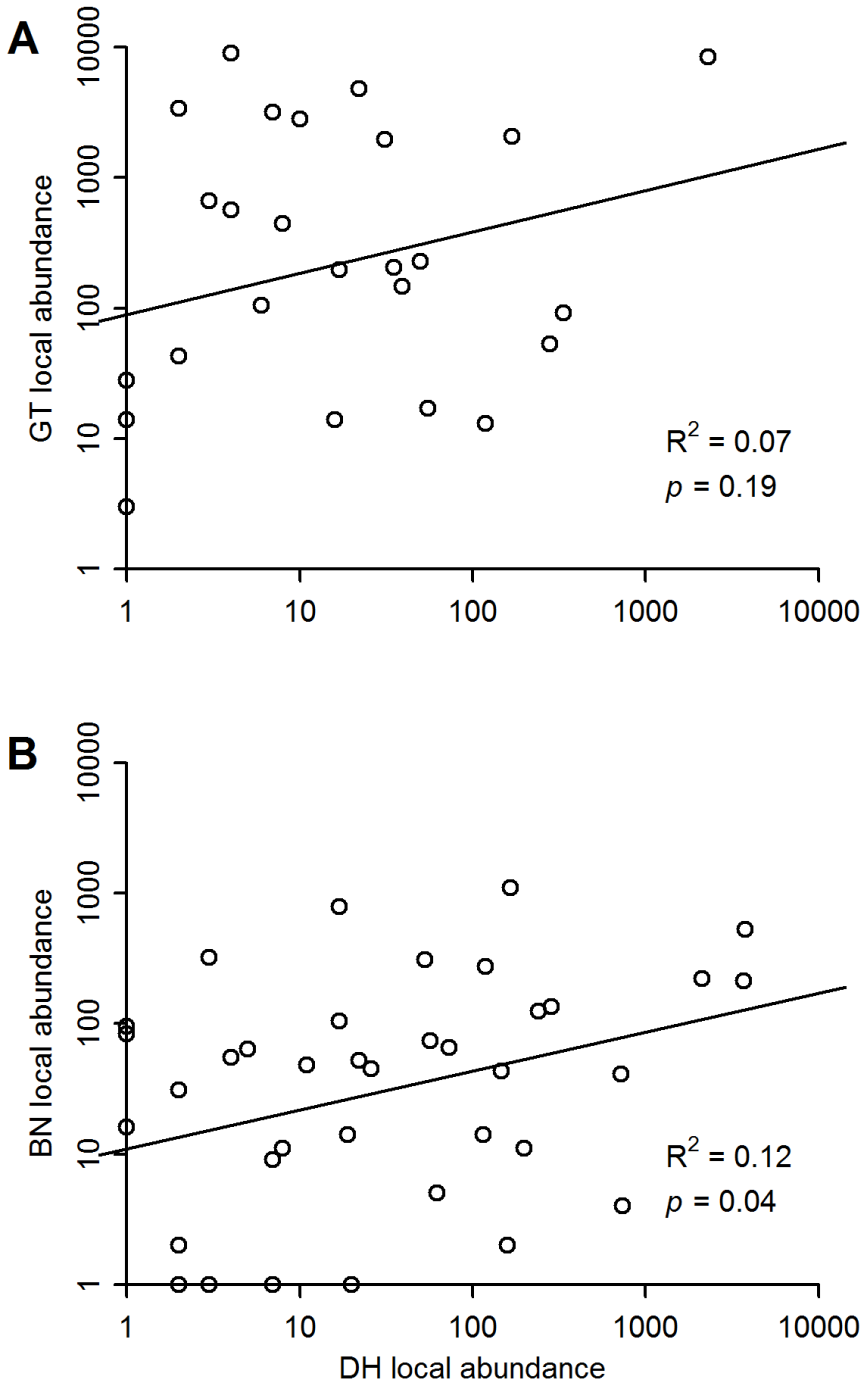
Correlation in local abundances of species between plots. Panel A is for Gutianshan (GT) vs. Dinghushan (DH). Panel B is for Xishuangbanna (BN) vs. Dinghushan. The axes are both log_2_-transformed. Lines are linear regressions and statistics from the Pearson correlation coefficients.

Species abundances were positively associated between sites (Fig. 7), significantly so when comparing Dinghushan and Xishuangbanna (R^2^ = 0.12, *p* = 0.04, *t = *2.17), but not significantly for the Gutianshan-Dinghushan comparision (R^2^ = 0.07, *p* = 0.19, *t = *1.36).

## Discussion

A wide majority of the 651 Chinese tree species we studied were widely distributed, while just under 10% had ranges <10^5^ km^2^ in China. All 63 of these range-restricted species were from the plots at Xishuangbanna and Dinghushan, near China’s border, and apart from those 18 species known to be endemic to China, they may have wide ranges to the south. The results are consistent with several studies of tree distributions [50]–[52]. For instance, Condit et al. [51] demonstrated that only 9.6% of the tree species in the forest plots around Panama Canal were endemic to Panama and Costa Rica, meaning their ranges were <1.3×10^5^ km^2^.

Included in our study were over 100 tree species endemic to China, whose entire ranges we now know. The endemics from Xishuangbanna, the southern tropical plot, had the smallest range sizes. This part of southeast of Yunnan province is a relic of an ancient flora and is known as a center of endemism [53], [54]. The result could also be taken as support of Rapoport’s rule [55], that low-latitude species have narrower ranges than high-latitude species.

We found no support for the biogeographic ‘law’ that abundance and geographic range are positively correlated, and indeed our results opposed the pattern [4], [23]. Our test rested on a single site per species where abundance was estimated, in contrast to much other works where local abundance was averaged over many sites across a species’ ranges [23], [56]. Nonetheless, the method we used for local abundance is often utilized [23], [57], [58], and the results from the two methods should coincide on average, unless the single site per species is biased regarding species’ optimal habitats [23], [57]. We see no reasons to expect this bias: three plots we studied are in subtropical lowland forest, and their environmental conditions are similar to the entire subtropical area. Our results are consistent with the few studies on tree species we found: some reported no correlation between local abundance and range size [25], and others found either positive or negative, though weak relationships [50], [59].

We did find consistent positive correlations in abundance between species across sites (Fig. 7), even those >1000 km apart (Fig. 1). Spatial concordance in abundance would result from consistent environmental conditions across sites [23], [57]. As discussed above, the plots we studied are all in lowland forest, two in the subtropics and the third at the edge of subtropics and tropics. None have unusual or specialized soils or climate. Our conclusion is thus that local abundance must be to some extent governed by species’ adaptations to the niches of subtropical moist forest. If populations were drifting due to strictly neutral processes [60], abundances at great distances should not be positively correlated. Nevertheless, there were substantial differences in abundance for many species, as is often found [27], [61].

A second biogeographic ‘law’ we tested was the abundance-center pattern, and our results did not support the notion that species are more abundant near their range centers. The intra-specific test we carried out clearly rejected the hypothesis, yet it was based on only two sites per species, and thus had limited power. Our second test was an inter-specific test, asking whether abundance differences among species are predicted by how far each is from it range center. There was no relation, consistent with the result from Williams *et al*. [25]. Evidently other factors accounting for species differences in abundance are much more important than the location of a range center.

Our analyses have implications for biodiversity conservation. First, the observation that most Chinese tree species we studied have large ranges suggests that extinction risks inherent in narrow geographic distribution [9] are not a major concern. Moreover, there is added risk to narrow-range species if they are also locally rare [7], [62]. The absence of a relationship between abundance and range suggests that Chinese tree species do not generally suffer the double extinction jeopardy of low population size and restricted range. Some species, however, are geographically restricted and locally rare, such as *Clerodendron trichotomum* at Gutianshan and *Cinnamomum mollifolium* at Xishuangbanna, and these need to be highlighted in identifying conservation needs [6], [10], [62]. A big step forward in documenting conservation priorities will be to merge our data from China with records from other Asian countries, in order to understand full range sizes. Meantime, we are increasing the number of plot sites across Chinese forests in order to better assess variation in local abundance [33].

## Supporting Information

Figure S1 **Correlation between range sizes fitted by a bi-variate Gaussian and by a minimum convex polygon (MCP).** Included are all 651 tree species with ≥20 geo-referenced records. Both axes were log2-transformed. The straight line is the regression line.
(TIF)

Method S1 **Procedure for running the Gibbs sampler to estimate range of species based on geo-referenced records.**
(DOCX)

Program S1 **Functions written in the R programming language for running a Gibbs sampler to estimate range sizes of species.**
(R) Click here for additional data file.
