# Supplementary figures and images for: Geographical Range and Local Abundance of Tree Species in China
Source: PLoS One. 2013 Oct 10;8(10):e76374. doi: 10.1371/journal.pone.0076374 (PMC3794993; doi:10.1371/journal.pone.0076374)

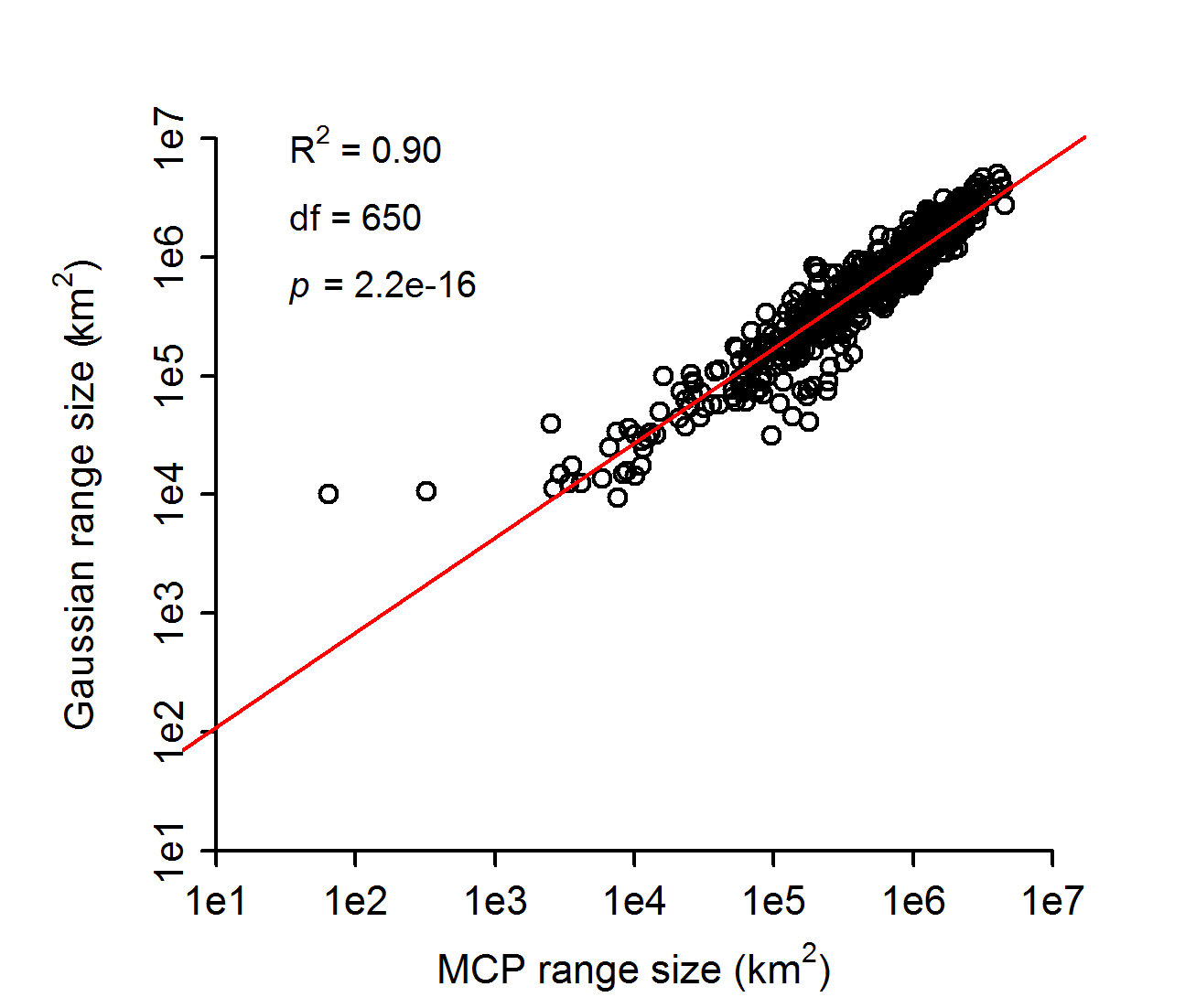

Supplement: Figure S1 — Correlation between range sizes fitted by a bi-variate Gaussian and by a minimum convex polygon (MCP). Included are all 651 tree species with ≥20 geo-referenced records. Both axes were log2-transformed. The straight line is the regression line. (TIF) [file pone.0076374.s001.tif]
