# Supplementary material for: Geographical Range and Local Abundance of Tree Species in China
Source: PLoS One. 2013 Oct 10;8(10):e76374. doi: 10.1371/journal.pone.0076374 (PMC3794993; doi:10.1371/journal.pone.0076374)
Supplement: Method S1 — Procedure for running the Gibbs sampler to estimate range of species based on geo-referenced records. (DOCX) [file pone.0076374.s002.docx]

Method S1. Procedure for running Gibbs sampler to estimate range of species based on geo-referenced records.

We assumed that $(x,y)\sim N_{2}\left(\mu,\Sigma\right)$, where $\mu=\left(\mu_{x},\mu_{y}\right)$, $\mu_{x}$ is the mean of the variable *x*, while $\mu_{y}$ is the mean of the variable *y*; $\Sigma=\left(\begin{matrix} \sigma_{x}^{2} & \mathrm{cov}_{xy} \\ \mathrm{cov}_{xy} & \sigma_{y}^{2} \end{matrix}\right)$, $\sigma_{x}^{2}$ is the variance for the variable *x*, $\sigma_{y}^{2}$ the variance for the variable *y*, and $\mathrm{cov}_{xy}$ the covariance between the variable *x* and *y*. We used Gibbs sampler to fit the hyper-parameters: $\mu_{x}$, $\mu_{y}$, $\sigma_{x}^{2}$, $\sigma_{y}^{2}$ and $\mathrm{cov}_{xy}$, and then generated the probability, $P_{i}\left(x,y\right)$, that a species *i* would be found at any location (*x, y*). The Gibbs sampler is based on the assumption that the prior distribution of each parameter is non-informative, that is uniform over all allowed values. The procedure to implement the Gibbs sampler was described as below.

Step 1. Assign initial hyper-parameter values: $\mu_{x\left[0\right]}$, $\mu_{y\left[0\right]}$, $\sigma_{x\left[0\right]}^{2}$, $\sigma_{y\left[0\right]}^{2}$ and $\mathrm{cov}_{xy\left[0\right]}$for each species *i*. Observed quantities for each parameter served as good starting points. The output of the Gibbs sampler beyond burn-in phase is insensitive to initial values.

Step2. Calculate the probability of $\mu_{x\left[0\right]}$, $P\left(\mu_{x\left[0\right]}\left|\mu_{y\left[0\right]},\sigma_{x\left[0\right]}^{2},\sigma_{y\left[0\right]}^{2},\mathrm{cov}_{xy\left[0\right]}\right.\right)$, conditional on all the other parameters, that is, holding all other parameters constant. A Gibbs sampling is then taken on $\mu_{x}$, namely a $\mu'_{x}$is randomly draw from a normal distribution centered on $\mu_{x\left[0\right]}$ with a predetermined standard deviation (called step size). The probability of $\mu'_{x}$ conditional on all initial parameters but $\mu_{x\left[0\right]}$, , is calculated. If the conditional probability of $\mu'_{x}$ is larger than that of $\mu_{x\left[0\right]}$, $\mu'_{x}$ is accepted as $\mu_{x\left[1\right]}$. If $\mu'_{x}$ is rejected, $\mu_{x\left[1\right]}$ = $\mu_{x\left[0\right]}$.

Step 3. Identical to step 2, but for the parameter $\mu_{y}$instead of $\mu_{x}$. This step is to produce the second value of $\mu_{y}$, $\mu_{y\left[1\right]}$, holding all other parameters constant. The value for the parameter$\mu_{x}$is its new value $\mu_{x\left[1\right]}$, while the rest are still at their first or initial values.

Step 4 etc. Repeat the same procedure for each of the rest parameters. The MCMC chain then includes two values for all parameters.

Then the process starts over at step 1 with updated $\mu_{x\left[1\right]}$, $\mu_{y\left[1\right]}$, $\sigma_{x\left[1\right]}^{2}$, $\sigma_{y\left[1\right]}^{2}$ and $\mathrm{cov}_{xy\left[1\right]}$, producing $\mu_{x\left[2\right]}$, $\mu_{y\left[21\right]}$, $\sigma_{x\left[2\right]}^{2}$, $\sigma_{y\left[2\right]}^{2}$ and $\mathrm{cov}_{xy\left[2\right]}$, and so on. Totally, we ran 1500s of these Gibbs steps. The first 500s were determined as burn-in period, and the final 1000s were used to estimate the five parameters and their credible intervals.
